# ECG low QRS voltage and wide QRS complex predictive of centenarian 360-day mortality

**DOI:** 10.1007/s11357-016-9907-0

**Published:** 2016-04-02

**Authors:** Jan Szewieczek, Zbigniew Gąsior, Jan Duława, Tomasz Francuz, Katarzyna Legierska, Agnieszka Batko-Szwaczka, Beata Hornik, Magdalena Janusz-Jenczeń, Iwona Włodarczyk, Krzysztof Wilczyński

**Affiliations:** 1 Department of Geriatrics, School of Health Sciences in Katowice, Medical University of Silesia, Katowice, Poland; 2 Department of Cardiology, School of Health Sciences in Katowice, Medical University of Silesia, Katowice, Poland; 3 Department of Internal Medicine and Metabolic Diseases, School of Health Sciences in Katowice, Medical University of Silesia, Katowice, Poland; 4 Department of Biochemistry, School of Medicine in Katowice, Medical University of Silesia, Katowice, Poland; 5 Department of Internal Nursing, School of Health Sciences in Katowice, Medical University of Silesia, Katowice, Poland

**Keywords:** Centenarians, ECG, Low QRS voltage, Left ventricular hypertrophy

## Abstract

We examined the electrocardiographic (ECG) findings of centenarians and associated them with >360-day survival. Physical and functional assessment, resting electrocardiogram and laboratory tests were performed on 86 study participants 101.9 ± 1.2 years old (mean ± SD) (70 women, 16 men) and followed for at least 360 days. Centenarian ECGs were assessed for left ventricular hypertrophy (LVH) according to the Romhilt–Estes score, Sokolow–Lyon criteria and Cornell voltage criteria which were positive for 12.8, 6.98, and 10.5 % of participants, respectively. Fifty-two study participants (60 %) survived ≥360 days. Multivariate logistic regression analysis revealed a negative relationship between 360-day survival and the following: R II <0.45 mV adjusted for CRP (odds ratio (OR) = 0.108, 95 % confidence interval (CI) = 0.034–0.341, *P* < .001), R aVF < 0.35 mV adjusted for CRP (OR = 0.151, 95 % CI = 0.039–0.584, *P* < .006), Sokolow–Lyon voltage <1.45 mV adjusted for CRP (OR = 0.178, 95 % CI = 0.064–0.492, *P* = .001), QRS ≥90 ms adjusted for CRP (OR = 0.375, 95 % CI = 0.144–0.975, *P* = .044), and Romhilt–Estes score ≥5 points adjusted for sex and Barthel Index (OR = 0.459, 95 % CI = 0.212–0.993, *P* = .048) in single variable ECG models. QRS voltage correlated positively with systolic and pulse pressure, serum vitamin B12 level, sodium, calcium, phosphorous, TIMP-1, and eGFR. QRS voltage correlated negatively with BMI, WHR, serum leptin, IL-6, TNF-α, and PAI-1 levels. QRS complex duration correlated positively with CRP; QTc correlated positively with TNF-α. Results suggest that Romhilt–Estes LVH criteria scores ≥5 points, low ECG QRS voltages (Sokolow–Lyon voltage <1.45 mV), and QRS complexes ≥90 ms are predictive of centenarian 360-day mortality.

## Background

Identification of electrocardiographic (ECG) findings predictive of centenarian survival would prove valuable in the clinical setting. The ECG remains one of the most ubiquitous diagnostic tools in medicine and is readily utilized for domiciled frail elderly patients. Survival prognosis of centenarians is especially important for making diagnostic and therapeutic decisions. Widespread specific cardiac anomalies have been documented by ECG in centenarians (Lakkireddy et al. 2003; Rabunal-Rey et al. 2012; Basile et al. 2012). Survival and ECG associations have been studied by Rabunal-Rey et al. (2012) who observed that atrial fibrillation is a prognostic factor for mortality and by Basile et al. (2012) who did not describe any ECG findings that are predictive of survival. While mildly elevated blood pressure has been associated with improved health status in centenarians (Szewieczek et al. 2011; Szewieczek et al. 2015a), consistently elevated blood pressure may lead to left ventricular hypertrophy (LVH). It follows then that a positive relationship between ECG LVH features and centenarian survival would be expected. However, LVH is a known risk factor for ischemic heart disease and death (Kannel et al. 1969; Kannel et al. 1970). Estes et al. (2015) demonstrated that the Romhilt–Estes score, traditionally used for ECG detection of LVH, may be applied to predict adverse outcomes in the general population. Despite poor correlation between electrocardiographic and echocardiographic features of LVH in centenarians (Martínez-Sellés et al. 2015), it seems reasonable to explore a possible association between ECG findings, especially LVH features, and survival probability in this specific age group. We also studied associations between ECG features and a range of clinical, metabolic, and inflammatory markers to ascertain possible influence on ECG features.

## Methods

### Participants

The study was carried out from January 2007 to August 2013. The data regarding >100-year-old inhabitants of Upper Silesia were obtained from the Silesian Voivodeship Administration Office in Katowice. In the year 2006, there were 389 centenarians living in Upper Silesia, accounting for 0.008 % of the total population of the area. Eight percent of addressees responded positively to invitation letters.

The study comprised of eighty-six 100.9 ± 1.2-year-old (mean ± SD) study participants (70 women and 16 men). The study group is defined in our previous papers (Szewieczek et al. 2015a, b).

### Measurements

Patient assessment comprised of a structured interview, physical examination, functional assessment, resting electrocardiography, and blood sampling. A detailed description of the methods used, beyond ECG analysis, is presented in our previous papers (Szewieczek et al. 2015a, b). Romhilt–Estes score, Sokolow–Lyon criteria, and Cornell voltage criteria were used to assess left ventricular hypertrophy (Buxton et al. 2006). An electrocardiogram (ECG) was analyzed according to the ACC/AHA/HRS Clinical Data Standards (Buxton et al. 2006) and considered normal if the following criteria were met: sinus rhythm, no significant rhythm disorders, heart rate from 60 to 90/min, electrical axis from −30 to 90, PR interval from 0.12 to 0.20 s, QRS complex duration from 0.06 to 0.11 s, corrected QT interval (QTc, according to the Bazett’s formula) ≤0.43 s in males, and ≤0.45 s in females, no significant ventricular depolarization or repolarization changes, negative Romhilt–Estes score, Sokolow–Lyon criteria, and Cornell voltage criteria. QTc >0.45 s in males or >0.46 s in females was considered prolonged QT. Centenarians received follow-up phone calls at least 360 days after initial examination.

### Statistical Analysis

Data was analyzed using STATISTICA software version 10 (StatSoft, Inc., USA; StatSoft Poland). Chi-square test, V-square test, and Fisher’s exact test were used for categorical variables, and nonparametric Mann–Whitney *U* test was used for quantitative variables to compare centenarians who survived 360 days with those who did not survive 360 days. Multivariate binary logistic regression was performed to assess electrocardiographic findings associated with 360-day survival. The variables were adjusted for clinical, functional, and laboratory factors. Multivariate analysis with backward elimination included variables that yielded *P* values of 0.1 or lower in the initial univariate analysis. The Kaplan–Meier method was used to estimate survival probability in subgroups of centenarians with respect to select variables, while differences between these subgroups were assessed with the Wilcoxon–Gehan statistic. Variables were tested for the value corresponding with the lowest *P* level. The nonparametric Spearman’s rank correlation coefficient was used to assess relationships between ECG, and clinical and laboratory measures. *P* values <0.05 were considered statistically significant.

### Ethics

The study protocol was approved by the Bioethical Committee of the Medical University of Silesia. Written informed consent was obtained from each participant and/or their caregiver after the aim of the study, protocol, and risks were explained.

## Results

Only seven study participants (8.14 %) presented with normal ECGs. Sixty-three participants (73.3 %) had sinus rhythm, 18 (20.9 %) had permanent atrial fibrillation, 1 had paroxysmal atrial fibrillation, 1 had paroxysmal atrial flutter, 1 had an implanted DDD pacemaker, and 2 had extrasinusal rhythms. Fifteen participants (17.4 %) presented with first-degree atrioventricular block, 17 (19.8 %) with right bundle branch block, and 4 (4.65 %) with left bundle branch block. Twenty-seven participants (31.4 %) had a prolonged QT interval. Electrocardiographic Romhilt–Estes score, Sokolow–Lyon criteria, and Cornell voltage criteria for LVH were positive for 12.8, 6.98, and 10.5 % of participants, respectively.

**Fig. 1:**
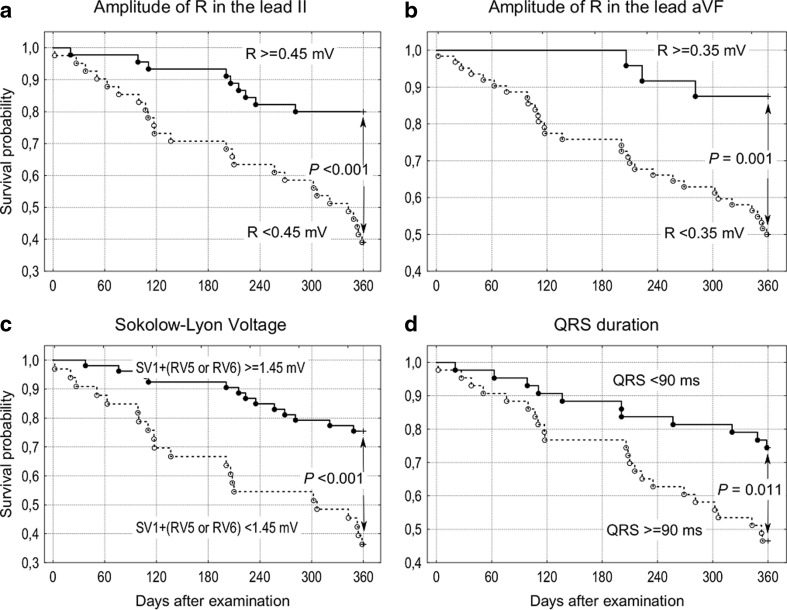
Kaplan–Meier survival probability of centenarians classified according to **a** amplitude of R wave in lead II ≥0.45 mV versus lower values, **b** amplitude of R wave in lead II ≥0.35 mV versus lower values, **c** Sokolow–Lyon voltage ≥1.45 mV versus lower values, and **d** QRS complex duration <90 ms versus higher values

Fifty-two participants (60 %) survived 360 days or more. The proportion of males who died (10 of 16) was greater than females (24 of 70; *P* = .038). Beyond sex, survivors, as compared to nonsurvivors, did not differ significantly in most clinical variables (see Table 1 in our previous paper: Szewieczek et al. (2015b)). Nonsurvivors, when compared to survivors, had a longer QRS complex, right ventricular activation time and QTc, lower R wave amplitude in leads II, aVF, V5, and V6, and lower S wave amplitude in lead V1 (Table 1). No significant differences were found with respect to the proportion of participants with positive LVH, as tested by the Sokolow–Lyon criteria, Cornell voltage criteria, or Romhilt–Estes score. Participants with an R wave in lead II < 0.45 mV, R wave in lead aVF <0.35 mV, Sokolow–Lyon voltage (S wave in lead V1 + R wave in lead V5 or V6—whichever is greater) <1.45 mV, and QRS complex duration ≥0.09 s had diminished 360-day survival probability as compared with study participants with higher or lower values, respectively (Fig. 1). Multivariate logistic regression analysis revealed a negative relationship between 360-day survival and the following: R wave in lead II <0.45 mV adjusted for CRP (odds ratio (OR) = 0.108, 95 % confidence interval (CI) = 0.034–0.341, *P* < .001), R wave in lead aVF <0.35 mV adjusted for CRP (OR = 0.151, 95 % CI = 0.039–0.584, *P* < .006), Sokolow–Lyon voltage <1.45 mV adjusted for CRP (OR = 0.178, 95 % CI = 0.064–0.492, *P* = .001), QRS ≥90 ms adjusted for CRP (OR = 0.375, 95 % CI = 0.144–0.975, *P* = .044), and Romhilt–Estes score ≥5 adjusted for sex and Barthel Index (OR = 0.459, 95 % CI = 0.212–0.993, *P* = .048) in single ECG variable models. QRS amplitude correlated positively with systolic blood pressure, pulse pressure and serum vitamin B12, sodium, calcium, phosphorous, and TIMP-1 levels, as well as with eGFR. QRS amplitude correlated negatively with BMI, WHR, and serum leptin, IL-6, TNF-α, and PAI-1. QRS complex duration correlated positively with CRP and bilirubin level, and negatively with eGFR. QTc interval correlated positively with TNF-α (Table 2).

**Table 1:** ECG measures in the study group

Variable	Survivors (*n* = 52)	Nonsurvivors (*n* = 34)	Survivors versus nonsurvivors
	Mean ± SD		*P*
QRS complex duration (s)	0.11 ± 0.11	0.12 ± 0.14	0.045
Ventricular activation time in V1 (s)	0.03 ± 0.04	0.05 ± 0.04	0.002
Corrected QT interval (s)	0.44 ± 0.07	0.45 ± 0.03	0.045
R wave in the lead II (mV)	0.56 ± 0.26	0.34 ± 0.25	<0.001
R wave in aVF (mV)	0.31 ± 0.22	0.18 ± 0.14	0.005
S wave in V1 (SV1) (mV)	0.77 ± 0.55	0.52 ± 0.49	0.020
R wave in V5 (RV5) (mV)	1.16 ± 0.56	0.88 ± 0.52	0.031
R wave in V6 (RV6) (mV)	1.05 ± 0.54	0.74 ± 0.42	0.012
Maximal R wave in V5 or V6 (mV)	1.22 ± 0.56	0.92 ± 0.51	0.023
SV1 + RV5 or RV6 (mV)	1.99 ± 0.72	1.44 ± 0.75	<0.001
Romhilt–Estes 5. criterion (1 point if QRS duration >0.09 s) (points)	0.38 ± 0.49	0.68 ± 0.48	0.023
Romhilt–Estes 5. criterion positive results (*n* (%))	20 (38.5)	23 (67.7)	0.008
Romhilt–Estes Score (points)	2.13 ± 2.09	3.00 ± 2.32	0.063
Romhilt–Estes Score positive results for probable LVH (LVH is likely with 4 or more points) (*n* (%))	10 (19.2)	13 (38.2)	0.052
Romhilt–Estes Score positive results for LVH (LVH is present with 5 or more points) (*n* (%))	4 (7.7)	7 (20.6)	0.079

**Table 2:** Correlations between clinical or laboratory measures and ECG measures in centenarians (*n* = 86)

Clinical and laboratory measures	ECG measures	Spearman’s rank correlation coefficient	*P* value
BMI (body mass index)	R wave in II	−0.213	0.049
	R wave in V5	−0.245	0.023
	R wave in V5 or V6	−0.233	0.031
	S wave in V1 + R wave in V5 or V6	−0.242	0.024
WHR (waist to hip ratio)	S wave in V1 + R wave in V5 or V6	−0.269	0.013
Systolic blood pressure	R wave in II	0.261	0.015
	R wave in aVF	0.307	0.004
	R wave in V5	0.223	0.039
	R wave in V5 or V6	0.262	0.015
	S wave in V1	0.250	0.020
	S wave in V1 + R wave in V5 or V6	0.261	0.015
Pulse pressure	Heart rate	−0.316	0.003
	R wave in aVF	0.239	0.026
	R wave in V6	0.304	0.004
	S wave in V1	0.316	0.003
	S wave in V1 + R wave in V5 or V6	0.335	0.002
CRP^a^	QRS complex duration	0.216	0.049
	Ventricular activation time in V1	0.302	0.005
Bilirubin^a^	QRS complex duration	0.256	0.019
Vitamin B12^a^	S wave in V1	0.254	0.022
Leptin^b^	R wave in V5	−0.331	0.030
Sodium^c^	R wave in II	0.327	0.025
Calcium^c^	R wave in II	0.400	0.004
	R wave in aVF	0.307	0.032
	R wave in V5	0.316	0.027
	R wave in V6	0.299	0.037
	S wave in V1 + R wave in V5 or V6	0.312	0.029
Phosphorus (inorganic)^c^	S wave in V1	0.447	0.001
	S wave in V1 + R wave in V5 or V6	0.383	0.007
	Cornell Voltage value (R wave in aVL + S wave in V3)	0.445	0.001
eGFR (CKD-EPI creatinine–cystatin C equation)	Heart rate	−0.273	0.012
	QRS complex duration	−0.237	0.030
eGFR (BIS_creatinine-cystatin C equation)	R wave in aVF	0.235	0.031
Interleukin 6^b^	R wave in V5	−0.328	0.028
	R wave in V6	−0.317	0.034
	R wave in V5 or V6	−0.333	0.025
	S wave in V1	−0.343	0.021
	S wave in V1 + R wave in V5 or V6	−0.526	0.000
Tumor necrosis factor alpha^b^	Corrected QT interval	0.307	0.040
Plasminogen activator inhibitor-1^b^	S wave in V1	−0.347	0.023
	Cornell Voltage value (R wave in aVL + S wave in V3)	−0.489	0.001
Tissue inhibitor of metalloproteinases 1^b^	Romhilt–Estes Score	0.313	0.041

^a^Serum level, assessed in 84 subjects

^b^Serum level, assessed in 43 subjects

^c^Serum level, assessed in 49 subjects

## Discussion

We found normal ECGs in 8.4 % of 86 study participants, which is consistent with other studies in the literature. Normal ECGs were observed in 5.7 % of 35 (Klich-Raczka et al. 2003), 7.6 % of 132 (Lakkireddy et al. 2003), 8.8 % of 80 (Rabunal-Rey et al. 2012), 16.7 % of 42 (Basile et al. 2012), and 26.2 % of 103 centenarians (Martínez-Sellés et al. 2015) in different study participants.

Commonly observed ECG abnormalities in our study group included atrial fibrillation, conduction defects, ST-segment depression, and left axis deviation. Permanent atrial fibrillation in our patients (20.9 %) occurred more frequently than observed by Klich-Raczka et al. (2003) (8.6 %) and Basile et al. (2012) (11.9 %), but less often than observed by Martínez-Sellés et al. (2015) (26.2 %), Rabunal-Rey et al. (2012) (26.3 %), or Lakkireddy et al. (2003) (29.5 %). Rabunal-Rey et al. (2012) demonstrated that atrial fibrillation is an independent risk factor for mortality. By contrast, Basile et al. (2012) did not find significant differences in age of death in relation to electrocardiographic findings. Lakkireddy et al. (2003) and Martínez-Sellés et al. (2015) did not report any relationship between ECG findings and mortality in centenarians.

Electrocardiographic assessment was positive for left ventricular hypertrophy in 6.98 to 12.8 % of participants in our study (depending on which ECG LVH assessment method was applied). In studies performed by Lakkireddy et al. (2003) and Basile et al. (2012), ECG features of LVH were found in 3 to 31 % of study participants. Martínez-Sellés et al. (2015) used echocardiography to diagnose left ventricular hypertrophy in 45 % of 100 centenarians, while only 5.1 % had ECG features of LVH. False-negative ECG-LVH may be associated with hypertension, increased systolic blood pressure, and prolonged QRS (Bacharova et al. 2015). These factors commonly exist in centenarians (Moubarak et al. 2012; Andersen-Ranberg et al. 2013; Szewieczek et al. 2015a; Szewieczek et al. 2015b).

Regardless of accuracy, ECG-LVH is associated with increased risk of cardiovascular events (Kannel et al. 1970; Bacharova et al. 2015; Porthan et al. 2015). Estes et al. (2015) found that four out of the six ECG components of the Romhilt–Estes score, beyond left axis deviation and prolonged QRS duration, were predictive of all-cause mortality in the general population. In previous studies, we observed a positive relationship between elevated blood pressure and 180-day survival (Szewieczek et al. 2015a) and between hypertension and 360-day survival (Szewieczek et al. 2015b) in centenarians.

Based on the association between QRS voltage and blood pressure (Van Hoof 1991), we expected a possible positive association between electrocardiographic LVH voltage criteria and survival in our study group. However, hypertension is a risk factor for left ventricular hypertrophy that is associated with an increased risk of ischemic heart disease, atrial fibrillation, heart failure, and mortality (Kannel et al. 1969; Kannel et al. 1970; Katholi and Couri 2011; Velagaleti et al. 2014; Cuspidi et al. 2015). Indeed, QRS voltage correlated positively with systolic blood pressure and pulse pressure (Table 2), and participants who survived 360 days had higher QRS voltage (Fig. 1).

Interestingly, LVH defined by a Romhilt–Estes score ≥5 in our study group was associated with decreased centenarian survival. This apparent contradiction may at least partially be explained by the high QRS voltage of the Romhilt–Estes LVH criteria (R wave amplitude greater than 2.0 mV in limb leads or R wave amplitude greater than 3.0 mV in leads V5 or V6) (Buxton et al. 2006). However, within our study population, we observed decreased survival probability at QRS amplitudes that were significantly lower than those suggested by the Romhilt–Estes criteria. Similarly, we found that the QRS amplitude cutoff for increased survival probability was also significantly lower (Fig. 1). These findings suggest that a positive Romhilt–Estes test for LVH and a low QRS voltage may be associated with decreased 360-day survival in our study group.

Low electrocardiographic QRS voltage (LQRSV) is traditionally defined by zenith-to-nadir QRS amplitudes of less than 0.5 mV in all limb leads and/or less than 1.0 mV in all the precordial leads (low limb, precordial, or total QRS voltages) (Madias 2008; Mussinelli et al. 2013). Etiologic factors include multiple cardiac, pericardial, and/or extra-cardiac causes (Madias 2008). LQRSV has been identified as a risk factor for increased mortality in individuals without apparent cardiovascular disease (Usoro et al. 2014) and in patients with acute coronary syndromes (Tan et al. 2015). Sokolow–Lyon voltage ≤1.5 mV is an alternative index of low QRS voltage (Austin et al. 2009). We were unable to prove an association between 360-day mortality and low QRS voltage in limb or precordial leads. Instead, we found such an association with low amplitude R waves in leads II and aVF, as well as a low Sokolow–Lyon voltage. The pathophysiology of the association between mortality and low amplitude of specific R waves in leads II and aVF is unclear.

Associations between mortality and decreased QRS voltage seem to be complex and may include common pathologies such as cardiomyocyte changes, diffuse myocardial fibrosis (Bacharova et al. 2015), and also amyloidosis which may be substantially more common than is currently recognized (Patel and Hawkins 2015). Sokolow–Lyon voltage correlates positively with left ventricular mass, left ventricular ejection fraction, and negatively with myocardial fibrosis in patients with hypertrophic cardiomyopathy (Konno et al. 2015).

Low QRS voltages were seen in 27.3 % of the light-chain (AL) amyloidosis patients and 12.9 % of the wild-type transthyretin amyloidosis (ATTRwt) patients (Pinney et al. 2013). ATTRwt, also known as senile cardiac amyloidosis, is an underdiagnosed disease that accounts for a significant number of elderly patients with heart failure with preserved ejection fraction (González-López et al. 2015). Low Sokolow–Lyon voltage is associated with a more severe myocardial infiltration in cardiac AL amyloidosis (Mussinelli et al. 2013) which can occur at any age (Patel and Hawkins 2015).

Study participants who did not survive 360 days as compared to survivors had a wider QRS complex, with increased right ventricular activation time. QRS widening is associated with increased risk for heart failure and mortality (Khan et al. 2007). The QRS interval can be affected by numerous factors, among them metabolic and inflammatory markers (Vanninen et al. 1996; Bacharova et al. 2012; Cetin et al. 2012). Focal or diffuse myocardial fibrosis, edema, inflammation, myocarditis, fatty replacement, or myocardial deposition of amyloid proteins may be determinants of myocardial tissue (Bacharova and Ugander 2014).

Inflammatory markers are elevated in patients with congestive heart failure and reflect disease severity (Lommi et al. 1997; Feldman et al. 2000). These observations may be substantiated by multiple associations between QRS voltage and various hemodynamic, metabolic, and inflammatory factors (systolic blood pressure, pulse pressure, serum vitamin B12, sodium, calcium, phosporus, TIMP-1 levels, and eGFR) found in our study, although elucidation of the significance of these relationships would benefit from further study.

Nonsurvivors as compared to survivors also had a longer QTc that correlated positively with TNF-α. QTc contributes independently to cardiovascular risk (Schouten et al. 1991). Kim et al. (2006) observed a relationship between CRP and QTc in middle-aged men and women. A similar relationship was observed in young, healthy men (Kazumi et al. 2003). Vasan et al. (2003) revealed an association between increased TNF-alpha, IL-6, and CRP levels in elderly patients and increased risk of congestive heart failure. Inflammation associated with rheumatoid arthritis was found to affect QTc (Panoulas et al. 2014).

Multiple metabolic and inflammatory factors may affect centenarian survival, influence heart structure and function, and therefore reflect characteristic ECG findings. These findings are generally consistent with other observations in the literature. As such, the importance of ECG characteristics as predictors of centenarian survival may signify more than simply a reflection of the cardiovascular health of the patient.

Criticism of the presented study encompasses low inclusion rate of candidates, which resulted in an extension of the observation period. Additionally, this study was based on a relatively small patient population. We were unable to complement our study with echocardiography that would substantially extend basis for data interpretation. These difficulties resulted mainly from the nature of the Polish centenarian cohort.

We demonstrated that a low QRS voltage and a wide QRS complex may be predictive of 360-day mortality among study participants. According to an extensive literature search, this is the first such published observation in centenarians. Since ECG remains one of the most accessible and noninvasive methods of health assessment, these findings may be useful for making diagnostic and therapeutic decisions in the oldest old patients.

## Conclusions

Results suggest that Romhilt–Estes LVH criteria scores ≥5 points, low ECG QRS voltages (Sokolow–Lyon voltage <1.45 mV), and QRS complexes ≥90 ms are predictive of centenarian 360-day mortality.
